# Transmission of Chronic Wasting Disease Identifies a Prion Strain Causing Cachexia and Heart Infection in Hamsters

**DOI:** 10.1371/journal.pone.0028026

**Published:** 2011-12-12

**Authors:** Richard A. Bessen, Cameron J. Robinson, Davis M. Seelig, Christopher P. Watschke, Diana Lowe, Harold Shearin, Scott Martinka, Alex M. Babcock

**Affiliations:** 1 Department of Immunology and Infectious Diseases, Montana State University, Bozeman, Montana, United States of America; 2 Department of Psychology, Montana State University, Bozeman, Montana, United States of America; 3 Department of Microbiology, Immunology, and Pathology, Colorado State University, Ft. Collins, Colorado, United States of America; University of Melbourne, Australia

## Abstract

Chronic wasting disease (CWD) is an emerging prion disease of free-ranging and captive cervids in North America. In this study we established a rodent model for CWD in Syrian golden hamsters that resemble key features of the disease in cervids including cachexia and infection of cardiac muscle. Following one to three serial passages of CWD from white-tailed deer into transgenic mice expressing the hamster prion protein gene, CWD was subsequently passaged into Syrian golden hamsters. In one passage line there were preclinical changes in locomotor activity and a loss of body mass prior to onset of subtle neurological symptoms around 340 days. The clinical symptoms included a prominent wasting disease, similar to cachexia, with a prolonged duration. Other features of CWD in hamsters that were similar to cervid CWD included the brain distribution of the disease-specific isoform of the prion protein, PrP^Sc^, prion infection of the central and peripheral neuroendocrine system, and PrP^Sc^ deposition in cardiac muscle. There was also prominent PrP^Sc^ deposition in the nasal mucosa on the edge of the olfactory sensory epithelium with the lumen of the nasal airway that could have implications for CWD shedding into nasal secretions and disease transmission. Since the mechanism of wasting disease in prion diseases is unknown this hamster CWD model could provide a means to investigate the physiological basis of cachexia, which we propose is due to a prion-induced endocrinopathy. This prion disease phenotype has not been described in hamsters and we designate it as the ‘wasting’ or WST strain of hamster CWD.

## Introduction

Chronic wasting disease (CWD) in deer and elk is an emerging prion disease of wildlife in North America. The natural cervid hosts for CWD include white-tailed deer (*Odocoileus virginianus*), mule deer (*Odocoileus hemionus hemionus*), Rocky Mountain elk (*Cervus elaphus nelsoni*), and moose (*Alces alces shirasi*) [1], [2]. The disease is characterized by a long incubation period of several years followed by the insidious onset of a progressive and fatal neurodegenerative disease [1]. Early symptoms in CWD can present as a wasting syndrome, characterized by a loss of body mass, and behavioral changes, both of which can span months in duration [1]. As CWD progresses a wide variety of additional symptoms and neurological disease have been observed and can include head tremors, ataxia, excessive salivation due to difficulty in swallowing, and aspiration pneumonia [1]. CWD is also a moderately contagious prion disease in both captive cervid farms and among free-ranging deer [3]–[5]. Prevalence rates of infection as high as 80% have been reported in captive farms among does and yearling white-tailed deer, and as high as 15% in free-ranging white-tailed bucks in the core endemic area of CWD in southern Wisconsin, but the prevalence is typically much lower [3]–[6]. Scrapie in certain breeds of sheep is another prion disease that can be contagious among sheep, but the other animal and human prion diseases are not thought to be transmissible under natural conditions [7]. The contagious nature of CWD and its more widespread geographic distribution than previously recognized has raised concerns that CWD, like bovine spongiform encephalopathy, may have zoonotic potential either through direct consumption of venison or indirectly by transmission to domestic animal species prior to consumption by humans. To date, the epidemiological and experimental data do not indicate that CWD is a zoonotic disease [8]–[10]. This should not be mistaken for definitive evidence that CWD is not zoonotic since the prevalence of CWD continues to increase among free-ranging cervids, and the extent of human exposure to CWD is difficult to estimate. The large number of susceptible cervids, moderate infection rates, and lack of effective strategies to control CWD transmission in wildlife has the potential to give rise to new biological strains whose pathogenicity for humans may be distinct from currently circulating CWD strains. As the percent of free ranging cervids with CWD increases, the number of humans exposed to CWD will likely also increase through consumption of venison from deer and elk with preclinical CWD. In the U.S. and Canada, diagnosis and management of CWD in wildlife should remain vigilant in order to minimize human exposure since once the first human case related to CWD were to become evident, it is likely that human exposure would be widespread due to the long incubation period observed in prion diseases.

CWD has been experimentally transmitted to non-cervid species in order to investigate interspecies transmission to domestic animals and non-human primates, and to establish CWD models in new host species. An efficient method to transmit CWD to rodents is to use PrP null mice lacking the endogenous murine prion protein gene (*Prnp*) in order to generate a transgenic mouse expressing *Prnp* from mule deer, white-tailed deer, or elk [9], [11]–[14]. These cervidized *Prnp* transgenic mice (TgMo-cerPrP) do not exhibit a prolonged incubation period upon interspecies CWD transmission compared to wild type mice, and have a similar microscopic pattern and brain distribution of the disease-specific prion protein, PrP^Sc^, as found in the brain of cervids with CWD [9], [11]. TgMo-cerPrP are also a valuable tool used to bioassay for CWD infectivity in tissues and fluids from CWD-infected cervids [15]. However, studies in TgMo-cerPrP do not report other disease characteristics that are specific to CWD in cervids including preclinical behavioral alterations, weight loss and a prolonged wasting disease, or PrP^Sc^ deposition in heart tissue. Although CWD transmission has been successful to many other host species including transgenic mice [9]–[11], [16], hamsters [16], [17], bank voles [18], ferrets [17], [19], mink [20], sheep [21], cattle [22], [23], and squirrel monkeys [24], [25], and results in a fatal neurodegenerative disease, many of the biological features distinct to CWD in cervids are not recapitulated upon interspecies transmission.

In the current study we investigated whether a model for CWD can be established in Syrian golden hamsters (SGH) that maintains key features of CWD in cervids. The rationale to pursue a SGH model was partially based on the ability to transmit many of the prion diseases (e.g., Creutzfeldt-Jakob disease, sheep scrapie, bovine spongiform encephalopathy, transmissible mink encephalopathy, and CWD) to SGH in order to investigate molecular and cellular mechanisms of disease. These studies have elucidated key aspects of prion pathogenesis including the physiochemical nature of the prion agent [26], the molecular basis of prion strain diversity [27]–[29], the physiological basis of prion-induced endocrine changes [30]–[32], routes of prion neuroinvasion [33], [34], the role of the lymphoreticular system in prion neuroinvasion [35], and the role of centrifugal prion spread in agent shedding [36], as well as many other biological features of prion diseases. To shorten the length of the incubation period previously reported upon interspecies transmission of CWD into SGH, we used a previously described approach in which CWD was inoculated into transgenic mice lacking the endogenous murine *Prnp* and overexpressing SGH *Prnp* using the rat neuron-specific enolase promoter (TgMo-sghPrP) [37], [38]. Serial passage of CWD into TgMo-sghPrP and subsequently into SGH resulted in a fatal neurodegeneration that was characterized by preclinical alterations in locomotor and behavioral activity and a prolonged wasting disease. In CWD-infected SGH the PrP^Sc^ distribution in brain had many similarities to CWD in cervids and the peripheral tissue distribution of PrP^Sc^ included the adrenal gland, pancreas, and heart, which are also characteristic of natural CWD [39]–[41]. We also observed an interesting prion distribution in the olfactory mucosa in which PrP^Sc^ deposition is primarily found at the border of the sensory epithelium and the lumen of the nasal airway, and we propose that this has implications for CWD shedding into nasal secretions and possibly for prion transmission. This study demonstrates that key features of CWD in cervids can be maintained upon interspecies passage and adaptation to the SGH and this model could be used to investigate the physiological basis of prion-induced wasting disease.

## Results

### Behavioral and clinical features of CWD in hamsters

To adapt CWD to rodents, we inoculated transgenic mice that express the SGH prion protein gene (*Prnp*), (TgMo-sghPrP), with two field isolates of CWD from clinical white-tailed deer. Subsequently, we inoculated SGH with CWD passaged into TgMo-sghPrP after one, two or three serial passages in order to adapt CWD to hamsters. We hypothesized that CWD should adapt faster upon interspecies and intraspecies passage in TgMo-sghPrP (i.e., shorter incubation periods) than SGH since they express approximately four-fold higher levels of SGH PrP^C^ than wild type SGH in the brain [16], [42]. With respect to *Prnp* genotype, experimental prion transmission between TgMo-sghPrP and SGH maintains the primary structure between the host PrP^C^ and infectious PrP^Sc^ and therefore, is analogous to an intraspecies transmission since this is the primary molecular determinant for efficient transmission between hosts [43]. One goal of this study was to establish CWD in SGH because this host species has been used to successfully investigate several prion diseases including scrapie [44], transmissible mink encephalopathy [27], [45], bovine spongiform encephalopathy [46], [47], and Creutzfeldt-Jakob disease [48]. However, a prion disease characterized by a progressive loss of body mass and cachexia has not been described in any of the prion diseases adapted to SGH even though it is a prominent feature of CWD in cervids. Table 1 summarizes a representative portion of the CWD transmission data from one of the white-tailed deer CWD isolates inoculated into TgMo-sghPrP and passaged into SGH. At first passage of CWD into four TgMo-sghPrP (recipient group M6148) the incubation period was 417±23.6 days. In Passage Line A one mouse from this group, M6148.1 (Table 1, see ‘Inoculation Number’), with an incubation period of 369 days was serially passaged into TgMo-sghPrP by intracerebal inoculation of a 1% (wt. vol.) brain homogenate and the incubation periods were 198±3.1 days at second serial passage (Table 1 recipient group M6298-99), 189±5.7 to 198±3.5 days at third serial passage (Table 1 recipient groups M6339 and M6337, respectively), and 187±1.8 days on the fourth serial passage (Table 1 recipient group M6386). The length of the clinical phase was very short in TgMo-sghPrP lasting approximately three days, and was characterized by ptosis, kyphosis, wasting, and hyperactivity. CWD passaged into TgMo-sghPrP from the first to third serial passages were also inoculated into SGH. The incubation periods upon first passage into SGH had a mean of between 355 and 389 days (Table 1 recipient groups H1429, H1470, H1471, H1569), which in one case was similar to the incubation period in one of the TgMo-sghPrP inocula (Table 1 inoculum number M6148.1 at 369 days), but much longer than the incubation periods in the other TgMo-sghPrP inoculum (Table 1 inoculum numbers M6298.1, M6299.1 and M6339.2; incubation periods ranging from 189 to 204 days). On second serial passage in SGH the mean incubation periods were slightly reduced and ranged from 322 to 343 days (Table 1 recipient groups H1570 and H1572), which remained significantly longer than the ∼200 day incubation period found in TgMo-sghPrP. The initial neurological symptoms in SGH infected with CWD were subtle and often it was difficult to assign a precise date for the onset of the clinical phase. Neurological symptoms of CWD in SGH included wasting, hyperactivity, a mild hyperexcitability when handled, an elongated horizontal posture during quadripetal motion, a mild impairment of hind limb motor function, and a circling behavior in the late stages of disease (Supplemental Video S1). Overall, serial passage of CWD into TgMo-sghPrP resulted in an average incubation period of between 187 to 198 days that when serially passaged into SGH resulted in a longer average incubation period between 322 and 389 days even though both rodent species have a similar *Prnp* genotype.

**Table 1:** Incubation period following serial passage of CWD isolate into transgenic mice and Syrian golden hamsters.

		TgMo(sgh-PrP)			Syrian golden hamster		
Recipient	Inoculum Number^2^	Incubation Period,			Incubation Period,		
Group^1^	(Incubation period)	days ± SEM	A/I^3^	Pass No.^4^	days ± SEM	A/I	Pass No.
M6148	CWD (na)	417±23.6	4/4	First			
**Passage Line A:**							
M6298-99	M6148.1 (369 d)	198±3.1	5/5	Second			
H1429	M6148.1 (369 d)				389±0	3/3	First
H1570	H1429.3 (389 d)				322±10.2	4/4	Second
M6337	M6298.1 (204 d)	198±3.5	5/5	Third			
H1470	M6298.1 (204 d)				373±3.1	4/4	First
M6339	M6299.1 (192 d)	189±5.7	4/4	Third			
H1471	M6299.1 (192 d)				379±3.0	4/4	First
H1572	H1471.4 (382 d)				343±5.0	8/8	Second
M6386	M6339.2 (189 d)	187±1.8	4/4	Fourth			
H1569	M6339.2 (189 d)				355±9.7	4/4	First
**Passage Line B:**							
M6332	M6148.3 (473 d)	360±16	3/3	Second			
M6374	M6332.1 (343 d)	160±4.6	4/4	Third			
M6388	M6374.4 (151 d)	157±0	3/3	Fourth			
H1604	M6374.4 (151 d)				477±15.4^5^	3/3	First

1 Recipient rodents were either TgMo(sgh-PrP) indicated by a ‘M’ preceding the Recipient Group number or Syrian golden hamsters indicated by a ‘H’ preceding the Recipient Group number.

2 Inoculum was either a 10% brain homogenate (CWD isolate and M6148.1 inoculation of hamsters), a 1% brain homogenate (all other passages except one), or a 0.01% brain homogenate (M6148.3) from an individual animal in the Recipient Group (indicated by .1, .2, .3, or .4). The incubation period of the transgenic mouse or Syrian golden hamster used as inoculum is indicated in the parenthesis. Inoculation of M6148.1 into recipient group H1429 was by the intra-olfactory bulb route as previously described.

3 A/I, number affected with prion disease versus total number inoculated.

4 Number of serial passages in either TgMo(sgh-PrP) or Syrian golden hamsters. Hamsters were inoculated with a CWD isolate that was serially passaged into TgMo(sgh-PrP) for one, two, or three times.

5 In recipient group H1604, one hamster was found dead at 456 days post-inoculation and a second exhibited characteristics of old age. They did not exhibit overt symptoms of wasting disease or neurological dysfunction, but brain from these two SGH were positive for PrP^Sc^. A third hamster was clinically positive for neurological disease and PrP^Sc^ in brain.

Although Passage Line A was representative of the majority of our findings on experimental transmission of CWD into TgMo-sghPrP, there were exceptions to this pattern. In Passage Line B, another transgenic mouse brain from recipient group M6148, M6148.3, which had an incubation period of 473 days, was initially passaged at a 10^−4^ dilution into TgMo-sghPrP. This resulted in an incubation period of 360±16 days for recipient group M6332 (Table 1). (This was different than Passage Line A in which i.c. inoculation of brain homogenates were performed at a 10^−2^ dilution.) On third and fourth serial passages at a 10^−2^ brain dilution, the incubation period shortened to 160±4.6 and 157±0 days, respectively (Table 1 recipient groups M6374 and M6388). Inoculation of third passage transgenic mouse M6374.4 brain (an incubation period of 151 days) into SGH resulted in an incubation period of 477±15.4 days even though overt neurological disease was only observed in one of three hamsters (Table 1 recipient group H1604). One hamster in this group was found dead at 456 days post-inoculation without any record of clinical symptoms, although it is possible that subtle symptoms of disease were missed. A second hamster was sacrificed at 468 days post-inoculation due to symptoms more consistent with old age. Analysis for PrP^Sc^ revealed high levels in the brain of both hamsters (data not shown). A third hamster in this group was culled at 520 days post-inoculation after a clinical course of several weeks. In this hamster terminal symptoms were characterized by a circling behavior, hyperactivity, and a loss of balance in the hind limbs when it would rear into an upright position. This disease was similar to hamster CWD from Passage Line A, but without the wasting syndrome. The long incubation period for recipient group H1604 was unexpected since the transgenic mouse used as inoculum (i.e., M6374.4 had an incubation period of 151 days) had a shorter incubation period than in TgMo-sghPrP used as CWD inoculum in Passage Line A (e.g., M6339.2 had an incubation period 189 days) in which transmission to SGH had an incubation period of 355±9.7 days (H1569.1) versus 477±15.4 days for H1604 (Table 1). These findings suggest that multiple CWD phenotypes can be isolated upon passage into TgMo-sghPrP, but additional studies are needed to determine whether these represent distinct CWD strains.

**Figure 1:**
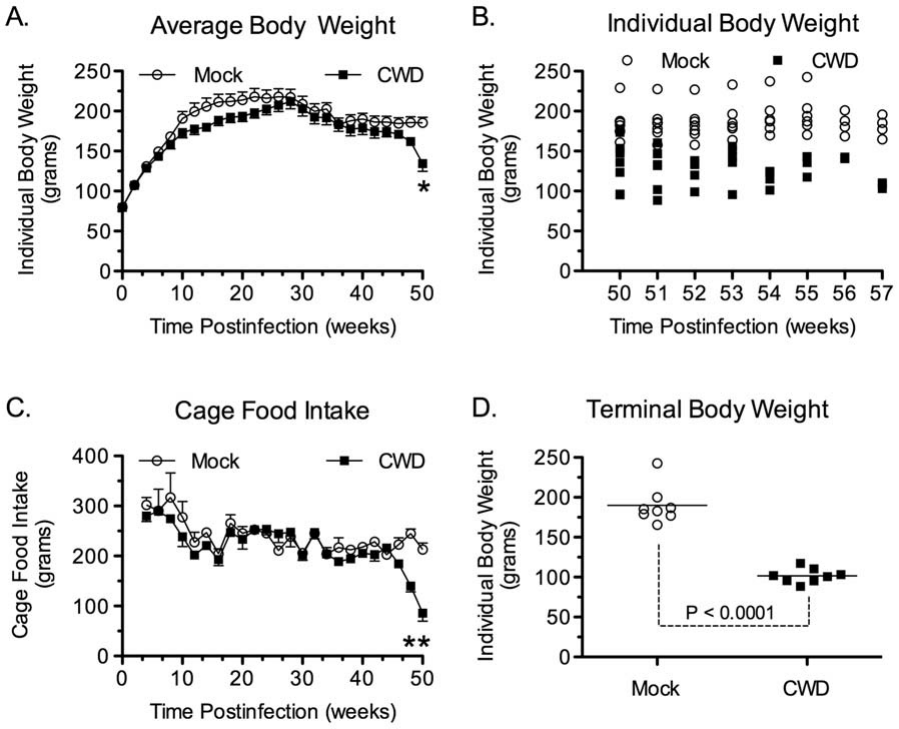
Time course of body weight and food intake in Syrian golden hamsters infected with chronic wasting disease. Mock infected (○) and CWD infected hamsters (▪) (see Table 1, recipient group H1572) were monitored weekly over the course of infection for individual body weight (A, B), cage food intake (C), and individual body weight at sacrifice (D). Values in panels (A) and (C) are the mean ± standard error of the mean, while in panels (B) and (D) the body weight is for individual SGH with the mean indicated by the solid horizontal bar (D). For time course data in (A) and (C), the curves for mock infected and CWD infected hamsters were compared by a mixed model ANOVA. In cases of a significant interaction, post hoc comparisons at each weekly time point were conducted using *t*-tests (corrected Bonferroni's *t*-test, * indicates p<0.001). Hamsters (n = 8 per treatment group) were sacrificed between 50 and 58 weeks post-inoculation (B). For illustration purposes (A, C), data points are plotted for every other week.

In SGH infected with CWD that was first passaged into TgMo-sghPrP in Passage Line A, a progressive loss of body mass was found beginning several weeks before onset of neurological symptoms. An analysis of individual hamster body weight versus time post-inoculation during the first 50 weeks post-inoculation indicates that there was a statistically significant treatment×time interaction (F(50,700) = 2.84, p = 0.0001; Figure 1A). Additional analysis revealed a statistical difference between mock and CWD hamster, but only at the 50-week time point (p<0.001, corrected Bonferroni's *t*-tests). Between 50 and 58 weeks post-inoculation hamsters with CWD continued to lose body weight and were culled when clinical symptoms progressed to the terminal stage of disease, thus excluding additional paired statistical analysis after 50 weeks post-inoculation (Figure 1B). There was also a significant treatment×time interaction in cage feed intake between mock and CWD hamster groups (F(48,96) = 3.64, p<0.0001; Figure 1C) and a significant decrease in cage feed intake in CWD-infected SGH from 48 to 50 weeks post-inoculation (Figure 1C; p<0.001, corrected Bonferroni's *t*-tests). This hypophagia was not due to an inability to access food and water. The loss of body mass and wasting disease was not initially accompanied by overt clinical symptoms and changes in neurological behavior were subtle for most of the clinical phase. At the time of sacrifice (mean of 54 weeks postinfection), the mean individual body weight for mock-infected SGH was 190±8.3 grams while for the CWD-infected SGH it was 102±3.2 grams, or a 45% reduction in average body weight (p<0.0001; Figure 1D). This reduction in body weight did not solely occur in the final few days before sacrifice, but was due to a progressive loss of body mass for individual hamsters over a mean duration of 7.25±1.1 weeks (Figure 1A & 1B; data not shown). This hypophagia and cachexia, or prolonged wasting disease phenotype, has not previously been described in SGH with prion disease and is designated as the ‘wasting’ or ‘WST’ strain of hamster CWD.

**Figure 2:**
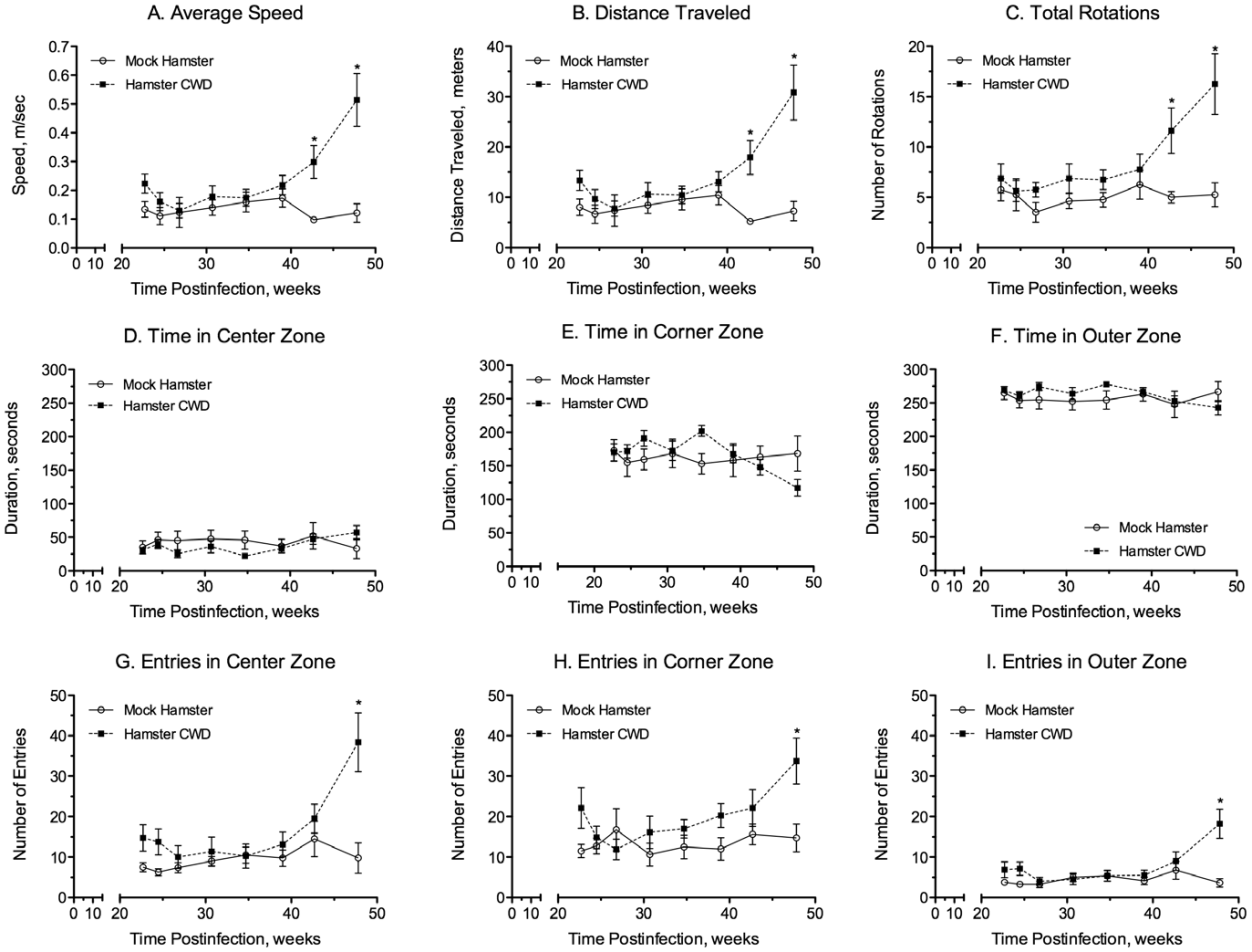
Open field behavior in Syrian golden hamsters infected with chronic wasting disease. Mock infected (○) and WST CWD infected hamsters (▪) (see Table 1, recipient group H1572) were monitored over the course of infection for locomotor activities including average speed (A), distance traveled (B), and total rotations (C) as well as thigmotaxis activities including time in the center (D), corner (E), and outer (F) zones, and the number of entries into the center (G), corner (H), outer (I) zones. Exploratory behavior of individual hamsters was measured using an automated tracking system (see Materials and Methods). Data for mock infected and CWD infected SGH were compared using a mixed model ANOVA and in the case of statistical significant interactions, a paired comparison was conducted using corrected Bonferroni's *t*-tests (* indicates p<0.001). For each treatment group n = 8 SGH.

The exploratory behavior of SGH infected with WST CWD passaged into TgMo-sghPrP was evaluated in an open field apparatus. This paradigm has been extensively used to evaluate locomotor activity patterns, anxiety and thigmotaxis, which refers to the tendency of rodents to remain close to the walls. Mock- and CWD-infected SGH (Table 1 recipient group H1572) were tested for three minutes every two to three weeks for a total of 19 weeks (between 29 and 48 weeks postinfection). The mean speed and distance traveled for the WST CWD-infected and control mock animals are depicted in Figure 2A and 2B. The locomotor activity of the two groups diverged at 43 weeks post-inoculation; CWD-infected SGH exhibited >3-fold increase in speed and distance compared to mock control SGH at that time point. Statistical analysis revealed a significant group×session interaction for both average speed (F(7,98) = 5.662, p<0.05) and distance traveled (F(7,98) = 5.737, p<0.05). Post-hoc analysis revealed that the average speed and distance traveled for CWD-infected animals were significantly higher compared to mock SGH at both 43 and 48 weeks post-inoculation (p<0.001, corrected Bonferroni's *t*-tests). The onset of changes in locomotor activity in WST CWD-infected hamsters occurred before statistically significant changes in hamster body weight, cage feed intake, and overt neurological symptoms of disease.

The behavior pattern of mock and WST CWD-infected SGH was also evaluated with respect to rotation behavior (i.e. 360° turn). Analysis of this locomotor activity revealed a significant treatment×time interaction (F(7,98) = 2.904, p<0.05) during the 19 weeks in which rotational activity was monitored and subsequent analysis showed a significant difference at 43 (p<0.01, corrected Bonferroni's *t*-tests) and 48 (p<0.001, corrected Bonferroni's *t*-tests) weeks post-inoculation (Figure 2C). Evaluation of thigmotaxis behavior revealed that the amount of time both groups spent in the outer zone of the apparatus (i.e., immediately adjacent to the wall) was significantly higher compared to the amount of time in the center zone across all testing sessions (Figure 2D, 2E, 2F). Animal behavior characterized by a propensity for areas close to walls (i.e., thigmotaxis) is a normal pattern of rodent behavior. No differences were observed between groups for the amount of time in the center, corner and outer zones (Figure 2D, 2E, 2F; p>0.05). Separate analysis revealed a significant treatment×time interaction for the number of entries into each of the designated zones of the apparatus (Figure 2G, 2H, 2I) and CWD-infected SGH entered all zones (i.e., center, corner, outer) with a greater frequency than mock-infected animals (p<0.05). These differences were only significant at 48 weeks post-inoculation (p<0.01, corrected Bonferroni's t-tests). At 48 weeks post-inoculation, WST CWD-infected SGH exhibited a 3.9-, 2.2- and 5.1-fold increase in the number of entries into the center, corner, and outer zones, respectively. These data indicate that the increase in locomotor activity observed in CWD-infected SGH was not preferentially distributed to any particular zone of the apparatus. The increase in the number of entries into each of the zones at 48 weeks post-inoculation in CWD-infected SGH may be partially due to the increase in locomotor activity (e.g., distance traveled, average speed, number of rotations) and hence, the number of times they travel through each zone.

### Neuropathological changes in CWD infection of hamsters

**Figure 3:**
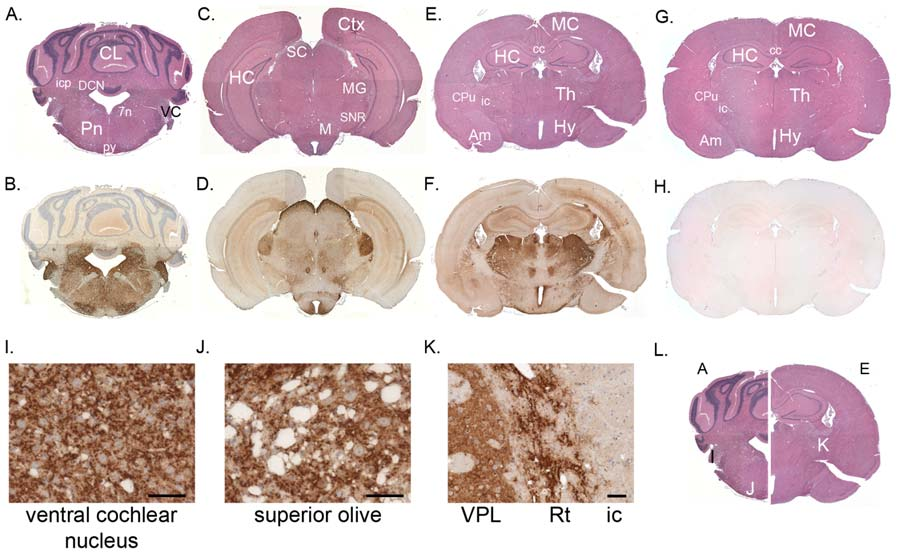
Brain distribution of PrP^Sc^ in Syrian golden hamsters infected with chronic wasting disease. Brain sections from WST CWD infected SGH (A through F and I through L) and mock-infected SGH (G, H) were stained with hematoxylin and eosin (A, C, E, G, L), or by PrP^Sc^ immunohistochemistry (brown deposit) counterstained with hematoxylin (B, D, F, H, I, J, K). Key: CL, cerebellum; DCN, deep cerebellar nuclei; Pn, pons; 7n, facial nerve; VC, ventral cochlear nucleus; py, pyramidal tract; Ctx, cerebral cortex; Am, amygdala; HC, hippocampus; SC, superior colliculus; MG, medial geniculate nucleus; SNR, substantia nigra; M, mammillary nucleus; MC, motor cortex; Th, thalamic nuclei, Hy, hypothalamic nuclei; CPu, caudate-putamen; icp, inferior cerebellar peduncle; ic, internal capusule; cc, corpus callosum; VPL, ventral posterolateral thalamic nucleus; and Rt, reticular thalamic nucleus. Scale bars in I through K is 50 µm.

To investigate whether neuropathological features of CWD in hamsters had similarities to CWD in cervids, the brain distribution of PrP^Sc^ in hamster WST CWD was evaluated by immunohistochemistry. The PrP^Sc^ brain distribution pattern was similar in all cases of hamster CWD for first and second passaged hamster CWD, even though there was some degree of animal-to-animal variation (Figure 3). The most consistent and intense PrP^Sc^ deposition in hamster CWD was observed in nuclei of 1) the forebrain particularly in the thalamus, hypothalamus, and to a lesser degree the hippocampus (Figure 3C to 3F and 3K); 2) the midbrain including the superior colliculus, substania nigra, and mammillary nucleus (Figure 3C and 3D); and 3) several areas in the caudal brainstem (Figure 3A, 3B, 3I, and 3J)(Table 2). Microscopically, the PrP^Sc^ immunostaining in hamster CWD brain varied from fine granular deposits to small aggregates to plaque-like deposits, with all three patterns found throughout affected brain regions (Figure 3I to 3K). PrP^Sc^ was primarily located in the extracellular neuropil, but intracellular deposits were sporadically observed particularly in the dorsal motor nucleus of the vagus.

**Table 2:** PrP^Sc^ distribution in the brain of hamsters infected with CWD.

Brain region	PrP^Sc^ in hamsters	PrP^Sc^ in cervids^1^
Forebrain:		
Cortical gray matter	+/++	+/++
Cortical white matter	0/+	+
Corpus callosum	0/+	+
Hippocampus	+/++	+
Hypothalamic nuclei^2^	++/+++	++/+++
Thalamic nuclei^3^	++/+++	+++
Amygdala	++	++
Striatum	+	++
Internal capsule	0/+	+
Midbrain:		
Superior colliculus	+++	++
Red nucleus	+	++
Substantia nigra	+/++	++
Oculomotor nucleus and tract	++	++
Mammillary nucleus	+++	++
Cerebellum:		
Molecular layer	0/+	+/++
Granular cell layer	0/+	++
Purkinje cell layer	0/++	++
Cerebellar white matter	+/++	+
Inferior cerebellar peduncle	0/+	0/+
Caudal Brainstem:		
Dorsal motor nucleus of the vagus	+++	+++
Raphe nuclei	++/+++	+/++
Reticular formation	++/+++	++
Solitary nucleus	++	++
Cochlear nucleus	+++	++
Vestibular nucleus	+++	+/++
Paraolivary nucleus	+++	+/++
Spinal trigeminal tract	0/+	++
Pyramidal tract	0/+	0/+
Other:		
Ependymal epithelium	++	++

The relative intensity of PrP^Sc^ deposition in the hamster brain infected with CWD was scored on a 0 to 3 scale with 0 = no immunoreactivity, 1 = mild immunoreactivity, 2 = moderate immunoreactivity, and 3 = intense immunoreactivity.

1 PrP^Sc^ distribution data in cervids was extrapolated from previously published reports and unpublished observations (D.M.S.) of terminal, CWD-infected captive mule deer and experimentally, orally-inoculated mule deer and white-tailed deer.

2 Hypothalamic nuclei that were evaluated include the arcuate, dorsomedial, supraoptic, paraventricular and subincertal nuclei.

3 Thalamic nuclei that were evaluated include the centrolateral, laterodorsal, lateral posterior, posterior, ventral posterolateral, posteromedial, ventromedial, paracentral, paraventricular, and mediodorsal nuclei.

To assess the similarities of the brain PrP^Sc^ deposition pattern in hamster CWD to cervid CWD, the global brain pattern of the PrP^Sc^ distribution in hamster CWD was compared to the PrP^Sc^ distribution in brain of cervids with CWD based on either prior publications [39], [49]–[52] or unpublished studies by one of us (D.M.S.). This PrP^Sc^ analysis demonstrated that there were many similarities between the two types of CWD in gray matter areas including those in the forebrain, midbrain, and brainstem, but less so in the cerebellum (Table 2). In contrast, there were small differences in the degree of PrP^Sc^ deposition within several major white matter tracts. In hamster CWD there was slightly less PrP^Sc^ deposition in cortical white matter, the corpus callosum, and spinal trigeminal tract, but slightly more severe in the cerebellar white matter compared to that reported for cervid CWD (Table 2). In other white matter tracts including the pyramidal tract, inferior cerebellar peduncle, and internal capsule, the PrP^Sc^ intensity was similar between hamster CWD and cervid CWD. At the cellular level the morphological nature of the PrP^Sc^ deposits were heterogenous and ranged from granular to plaque-like in CWD infections of both hamsters and cervids. Overall, the morphology and distribution of PrP^Sc^ in hamster WST CWD was largely indistinguishable from terminal, CWD-infected cervids.

### Distribution of peripheral infection in hamster CWD

**Figure 4:**
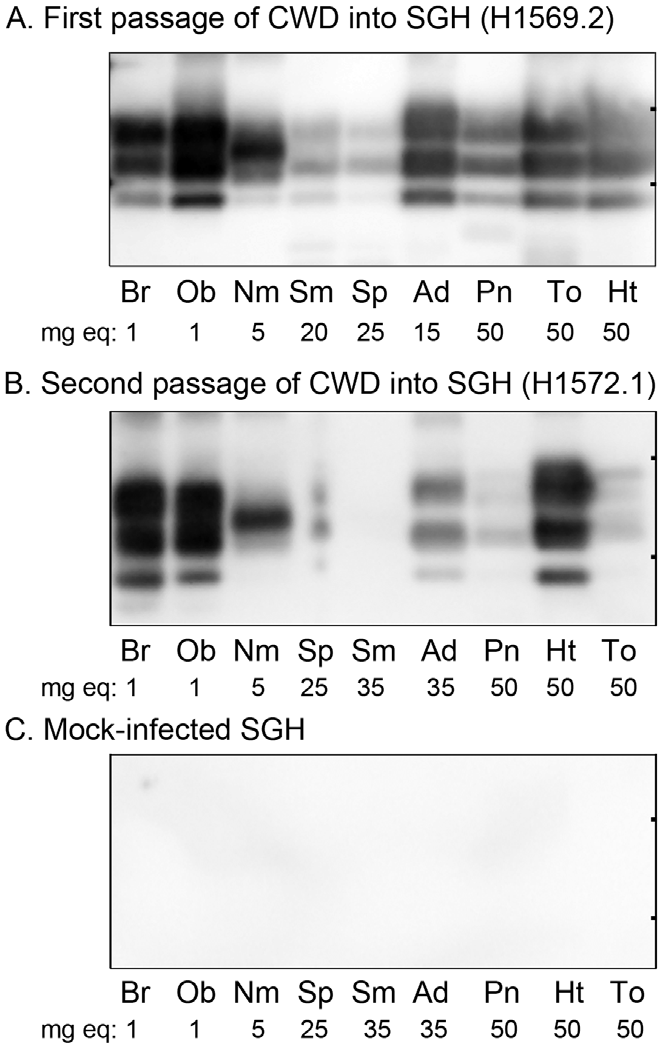
Western blot for PrP^Sc^ in central and peripheral tissues of Syrian golden hamsters infected with chronic wasting disease. Tissues from hamsters infected with WST CWD after first (A) and second (B) passage (see Table 1, recipient groups H1569 and H1572) and mock infected brain (C) were enriched for PrP^Sc^ by differential ultracentrifugation and proteinase K digestion followed by NuPAGE and anti-PrP western blot analysis. The amount of tissue equivalents in milligrams (mg eq) loaded into each lane is indicated below each tissue. Key: Br, brain; Ob, olfactory bulb; Nm, nasal mucosa; Sm, submandibular lymph node; Sp, spleen; Ad, adrenal gland; Pn, pancreas; To, tongue; and Ht, heart. Tick marks on the right vertical frame of each panel indicate the 20 and 30 kDa molecular weight markers.

To further investigate the biological relevance of CWD in hamsters to CWD infection in the natural deer host, we analyzed the distribution of PrP^Sc^ in the nervous, lymphoreticular, endocrine, and olfactory systems as well as in skeletal muscle and compared these results to those previously reported for the PrP^Sc^ distribution in cervids with CWD [15], [39]–[41], [49]–[51], [53]–[56]. Western blot revealed strong PrP^Sc^ deposition in the brain and olfactory bulb, moderate-to-high levels in the nasal mucosa, and lower levels in spleen and lymph nodes of SGH infected with CWD on first and second passage from TgMo-sghPrP (Figure 4A and 4B). This central and peripheral tissue distribution of PrP^Sc^ in WST CWD-infected SGH is also found in many prion diseases, while in the neuroendocrine system it has been mainly described in natural prion diseases of deer and sheep and infection in heart tissue has been described in deer and elk with CWD [39]–[41], [57], [58]. In WST CWD-infected SGH PrP^Sc^ was found in both the adrenal gland and pancreas with higher levels present in the adrenal gland. The adrenal gland had higher levels of PrP^Sc^ than both the spleen and submandibular lymph node in first passage in SGH (Figure 4A). PrP^Sc^ deposition was also found in the heart and tongue tissue with equivalent amounts on first passage, but higher levels in the heart on second serial passage in SGH although animal-to-animal variation was observed (Figure 4A and 4B). PrP^Sc^ deposition was not detected in tissues from the nervous, olfactory, peripheral endocrine, and musculature systems from mock-infected SGH (Figure 4C). In WST CWD-infected SGH the PrP^Sc^ deposition was consistent with the tissue distribution of PrP^Sc^ in deer with CWD, specifically with respect to the adrenal gland, pancreas, and heart tissue.

**Figure 5:**
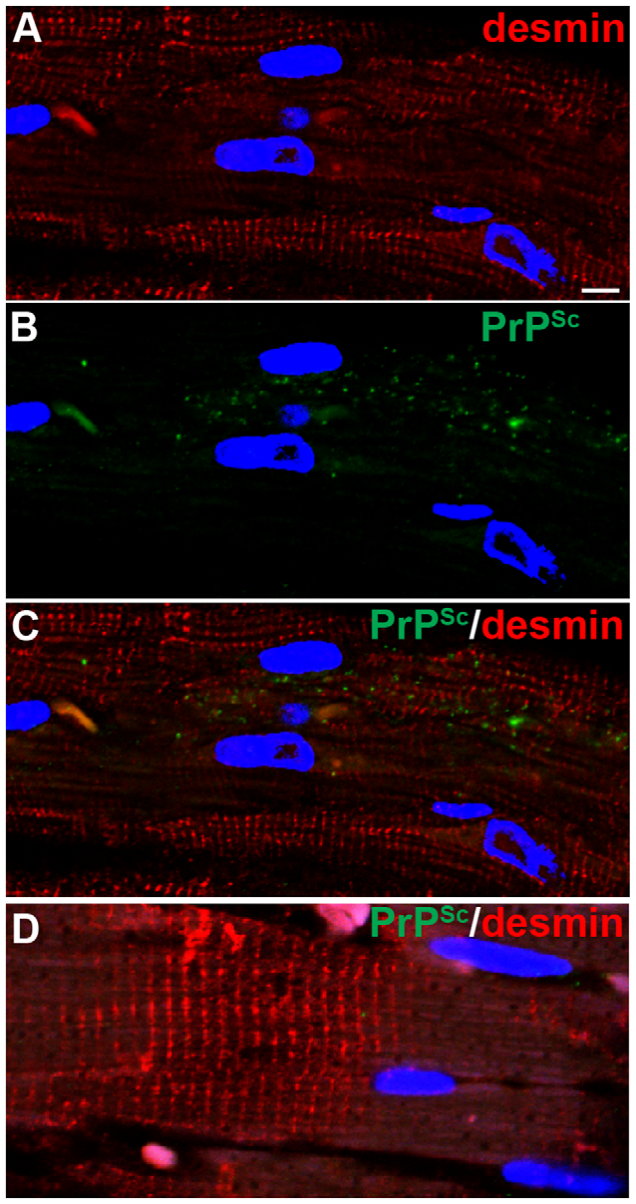
Immunohistochemistry for PrP^Sc^ in heart in Syrian golden hamsters infected with chronic wasting disease. Skeletal muscle of heart from mock (D) and WST CWD-infected (A through C) hamsters. Panels A through C are the same field of view that are separated into three panels according to the immunofluorescence staining. Heart was analyzed by dual immunofluorescence for desmin (A, C, D) and PrP^Sc^ (B, C, D). ToPro®-3 staining of nuclei is indicated by blue fluorescence. Scale bar in A is 10 µm.

**Figure 6:**
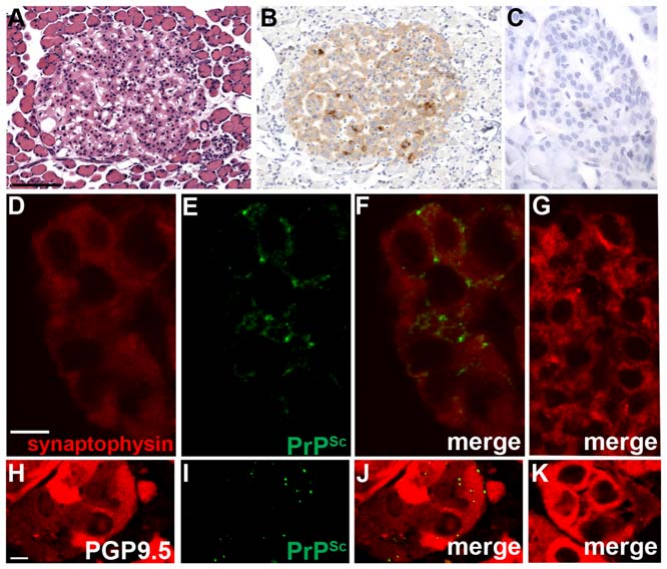
Immunohistochemistry for PrP^Sc^ in neuroendocrine tissues in Syrian golden hamsters infected with chronic wasting disease. Pancreas (A through G) and adrenal gland (H through K) from mock (C, G, K) and WST CWD-infected (A, B, D, E, F, H, I, J) hamsters. Panels D through F, and H through J are the same field of view, which are separated into three panels according to the immunofluorescence staining. Pancreas was analyzed by dual immunofluorescence for synaptophysin (D, F, G) and PrP^Sc^ (E, F, G) while the adrenal gland was analyzed by dual immunofluorescence for PGP9.5 (H, J, K) and PrP^Sc^ (I, J, K) using laser scanning confocal microscopy. Serial sections of pancreas were also analyzed by hematoxylin and eosin (A) and PrP^Sc^ immunohistochemistry (B, C; brown punctate aggregates) and counterstained with hematoxylin. Scale bar in D and H is 10 µm and in A the scale bar is 100 µm.

To further investigate the cellular distribution of PrP^Sc^ in the heart, pancreas, and adrenal gland in hamsters with WST CWD, PrP^Sc^ immunohistochemistry and dual immunofluorescence (IMF) was performed and compared to the cellular PrP^Sc^ distribution in these tissues in deer and elk with CWD as previously reported. To determine the distribution of PrP^Sc^ in heart, dual IMF was performed with PrP^Sc^ and desmin, a cytoskeletal protein in muscle cells. Desmin immunofluorescence was localized to the Z band of muscle cells in the heart and PrP^Sc^ deposits were found within desmin-positive cells indicating prion infection of cardiac muscle (Figure 5A to 5C). In the pancreas of hamsters with CWD, PrP^Sc^ was almost exclusively observed in the Islets of Langerhans as punctate deposits (Figure 6A and 6B). Dual IMF for PrP^Sc^ and synaptophysin in the pancreas of hamster CWD revealed that PrP^Sc^ deposition was limited to synaptophysin-positive structures, which we interpret as prion infection localized to neuroendocrine tissue in the Islets of Langerhans (Figure 6D to 6F). In the adrenal gland, PrP^Sc^ punctate immunofluorescence was found in the medulla of the adrenal gland and appeared restricted to PGP 9.5-positive structures (Figure 6H to 6J). This also indicates that PrP^Sc^ was found within the neuroendocrine portion of the adrenal gland. PrP^Sc^ deposits were not detected in the heart, pancreas, and adrenal gland from mock-infected hamsters (Figures 5D, 6C, 6G, and 6K). These findings demonstrate that PrP^Sc^ is localized to cardiac muscle and peripheral neuroendocrine tissues in hamster WST CWD and this is consistent with the PrP^Sc^ distribution in these tissues from deer and elk with CWD as previously published [39]–[41].

### Distribution of hamster CWD in the olfactory system

**Figure 7:**
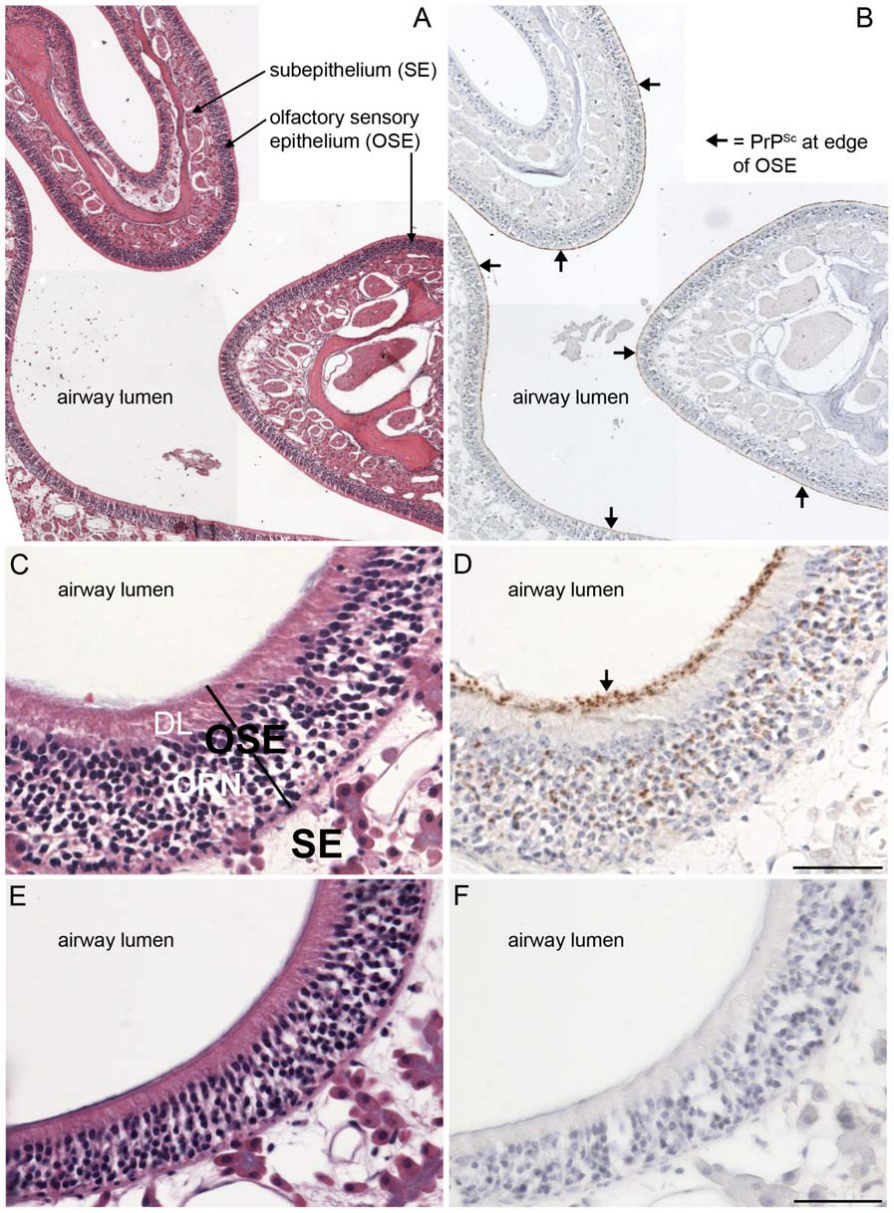
Distribution of PrP^Sc^ in nasal cavity in Syrian golden hamsters infected with chronic wasting disease. Low (A, B) and high (C, D, E, F) magnification photomicrographs of the nasal cavity from Syrian golden hamsters infected with WST CWD brain (A, B, C, D) and mock-infected brain (E, F) were stained with hematoxylin and eosin (A, C, E,) or immunostained for PrP^Sc^ (brown deposit) and counterstained with hematoxylin (B, D, F). Key: OSE, olfactory sensory epithelium; SE, subepithelial layer; ORN, olfactory receptor neurons; and DL, dendrite layer. Arrows indicate PrP^Sc^ deposition at the border of the OSE layer and lumen of the nasal airway. Scale bar in D and F is 50 µm.

Prion infection of the olfactory bulb and nasal mucosa previously has been described in sheep with scrapie [57]–[59], deer and elk with CWD [56], humans with sporadic Creutzfeldt-Jakob disease [60], [61], and hamsters with experimental prion disease [35], [62]. The olfactory mucosa has been proposed to be a site of prion shedding since olfactory receptor neurons at this site can replicate prions to high levels and prion infectivity is found in nasal fluids [36], [63]. In CWD infected SGH PrP^Sc^ was detected in homogenates of the olfactory bulb and nasal mucosa at both the first and second serial passages as described above (Figure 4A and 4B). The molecular weight of proteinase K digested PrP^Sc^ from the nasal mucosa was characterized by a major PrP^Sc^ polypeptide at approximately 27 kDa, this PrP^Sc^ polypeptide pattern was distinct from that in the olfactory bulb and brain. This major PrP^Sc^ polypeptide in the nasal mucosa extracts had a size that was between the diglycosylated and monoglycosylated PrP^Sc^ polypeptides found in the central nervous system and other peripheral tissues. Using immunohistochemistry PrP^Sc^ was found in the olfactory bulb, but primarily in the glomerular layer and outer plexiform layer on both first and second passage of WST CWD into SGH (Table 3). In the nasal cavity, PrP^Sc^ was prominent in the olfactory sensory epithelium (OSE), but not in nerve fibers in the subepithelial layers or in the non-sensory epithelium on both first and second serial passage in SGH (Figure 7A to 7D, Table 3). Within the OSE the strongest PrP^Sc^ staining was observed along the edge of the OSE at the interface with the lumen of the nasal airway in a location consistent with the distal knob of the dendrites projecting from olfactory receptor neurons (ORNs). Often this PrP^Sc^ deposition pattern was continuous along the length of the OSE border (Figure 7A to 7B, Table 3). A lower amount of PrP^Sc^ was observed in the dendrite layer of ORNs, while moderate PrP^Sc^ staining was observed in the cell layer of the OSE, which contains the cell bodies of ORNs (Figure 7C and 7D, Table 3). Mock-infected hamsters did not exhibit immunostaining for PrP^Sc^ in the nasal mucosa (Figure 7E and 7F).

**Table 3:** PrP^Sc^ distribution in nasal cavity and olfactory bulb.

	Olfactory sensory epithelium			Subepithelium^1^		Olfactory bulb^2^				
Hamster	Cell body	Dendrite	Knob	Nerve bundle	NALT	ONL	GL	OPL	M	IPL
H1470.2	0.9^3^	0.0	2.1	0.0	0.0	nd	nd	nd	nd	nd
H1470.3	0.0	0.0	0.9	0.1	0.0	0.0	0.9	0.3	0.0	0.0
H1471.2	0.6	0.0	1.6	0.0	0.0	0.0	0.8	0.5	0.0	0.0
H1471.3	0.3	0.0	1.2	0.0	0.0	0.0	1.1	0.5	0.0	0.0
H1472.1	1.4	0.2	2.9	0.5	0.0	0.0	3.0	2.2	0.0	0.0
H1472.3	0.5	0.0	1.5	0.0	0.0	0.0	0.7	0.8	0.4	0.0
Average:	0.6	<0.1	1.7	<0.1	0.0	0.0	1.3	0.8	<0.1	0.0
SEM:	0.2	<0.0	0.3	<0.1	0.0	0.0	0.5	0.3	<0.1	0.0

1 Subepithelium of nasal mucosa; NALT, nasal-associated lymphoid tissue.

2 Olfactory bulb structures include the ONL, outer nerve layer; GL, glomerular layer; OPL, outer plexiform layer, M, mitral layer; IPL, inner plexiform layer.

3 The relative intensity of PrP^Sc^ deposition in the hamster brain infected with CWD was scored on a 0 to 3 scale with 0 = no immunoreactivity, 1 = mild immunoreactivity, 2 = moderate immunoreactivity, and 3 = intense immunoreactivity. For each hamster a minimum of twenty-four tissue sections were examined spanning the middle to posterior portion of the nasal cavity.

nd, not done.

**Figure 8:**
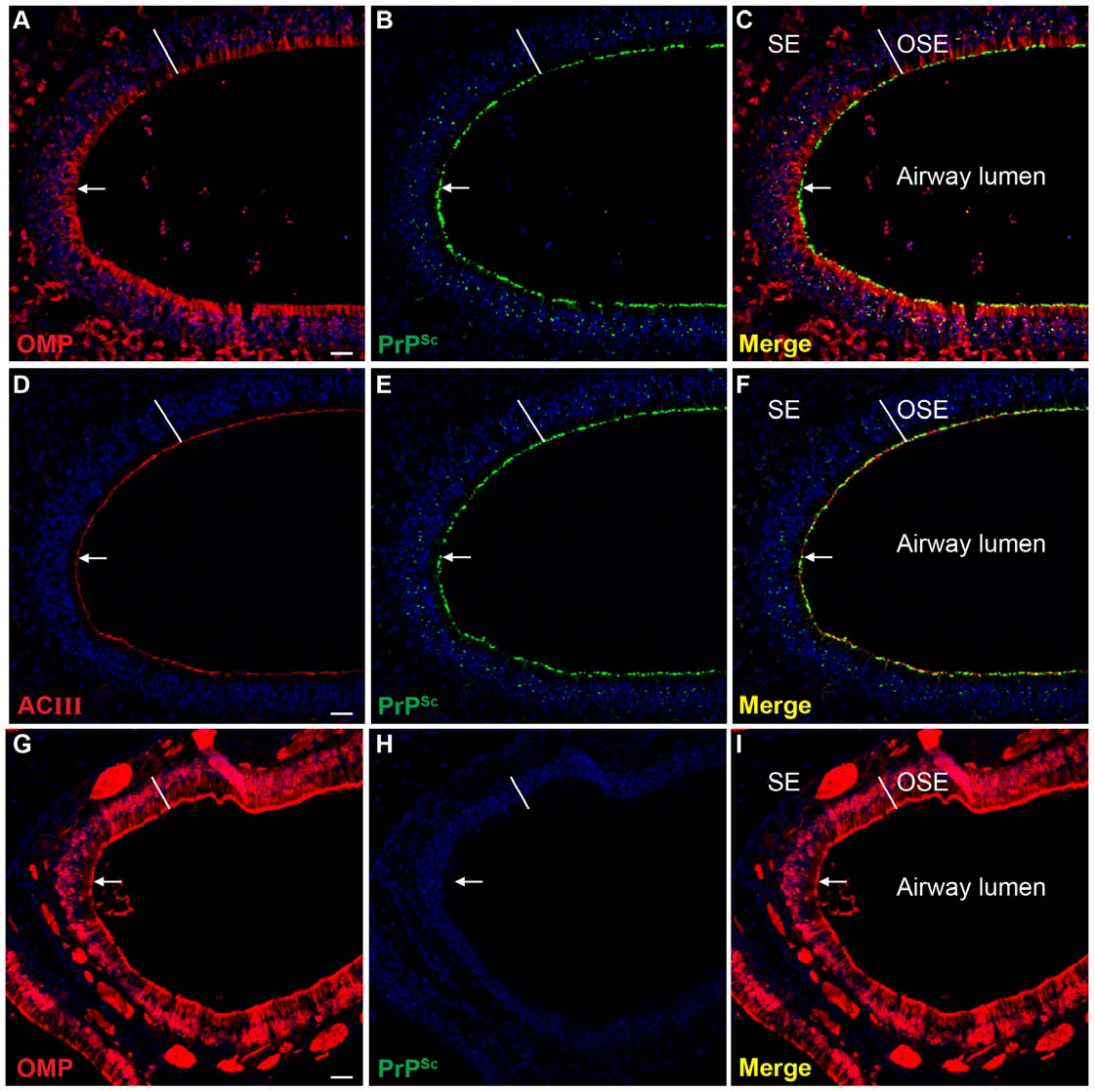
Distribution of PrP^Sc^ in olfactory sensory epithelium in Syrian golden hamsters infected with chronic wasting disease. Laser scanning confocal microscopy of olfactory marker protein (OMP)(*A, G*), PrP^Sc^ (*B, H*), and for both OMP and PrP^Sc^ (Merge)(*C, I*) in Syrian golden hamsters infected with TgMo-sghPrP CWD (A, B, C) and mock-infected hamsters (G, H, I). Laser scanning confocal microscopy of adenylyl cyclase III (ACIII)(*D*), PrP^Sc^ (*E*), and for both ACIII and PrP^Sc^ (Merge)(*F*) in CWD infected SGH. Panels *A* through *C*, *D* through *F* and G through I are the same field of view that are separated into three panels according to the immunofluorescence staining. ToPro®-3 staining of nuclei is indicated by blue fluorescence. Olfactory receptor neurons in the olfactory sensory epithelium (OSE width indicated by white line), and nerve fibers in the subepithelial layer (SE), both express high levels of OMP (A, C and G and I). ACIII is located on the sensory cilia that project from the terminal dendrites of ORNs and its distribution was prominent at the border between the OSE and airway lumen (D and F). The white arrow points to the distal edge of the OSE where it borders the lumen of the nasal airway. Scale bar in A, D and G is 50 µm.

To further investigate the distribution of PrP^Sc^ in the OSE, laser scanning confocal microscopy (LSCM) was used to examine the spatial relationship of PrP^Sc^ to ORNs and the cilia of ORNs by dual immunofluorescence for PrP^Sc^ and either olfactory marker protein (OMP) or adenylyl cyclase III (ACIII), respectively. At low magnification the PrP^Sc^ distribution was continuous with the outer edge of the OMP-positive OSE layer while a lower level of PrP^Sc^ immunofluorescence was observed in the cell body layer of the OSE (Figure 8A to 8C), which was consistent with the PrP^Sc^ immunohistochemistry (Figure 7C and 7D). A three-dimensional reconstruction of the OSE at higher magnification revealed PrP^Sc^ deposition within OMP-positive ORNs with evidence for moderate amounts of PrP^Sc^ in cell bodies, lower amounts in the dendrites of ORNs, and intense PrP^Sc^ labeling at the distal dendrites of OMP-positive ORNs, which is adjacent to the lumen of the nasal airway (Supplemental Video S2). This last PrP^Sc^ deposition pattern could be associated with either dendritic knobs of the ORNs, cilia of ORNs, or both structures. LSCM of PrP^Sc^ and ACIII, which is located to the inner membrane of cilia that project from the dendritic knobs of ORNs into the mucus layer, revealed that the distribution of PrP^Sc^ at the edge of the airway lumen was closely aligned with the ACIII distribution (Figure 8D to 8F). A three-dimensional reconstruction of LSCM analysis of PrP^Sc^ and ACIII revealed that the PrP^Sc^ pattern at the border between the OSE and airway lumen was also consistent with a distribution in the cilia of ORNs (Supplemental Video S3). Mock-infected SGH did not exhibit evidence of PrP^Sc^ deposition (Figure 8G to 8I). PrP^Sc^ deposition was not found in three-dimensional reconstructions of the OSE from mock-infected SGH when dual immunofluorescence was performed with OMP or ACIII (Supplemental Video S4). This continuous PrP^Sc^ deposition pattern along the edge of the OSE in WST CWD infected SGH may have implications for prion agent shedding into nasal secretions.

## Discussion

A prominent feature of CWD in deer is a progressive loss of body mass and adiposity over a period of weeks to months [1]. This observation is not unique among prion diseases since a wasting phenotype is also found in sheep and goats with scrapie [64]. Despite interspecies transmission of CWD and scrapie to rodents, a similar progressive wasting disease has not been described in SGH except in the terminal stages of disease [31]. In the current study, we describe a progressive loss of body mass and cachexia over several weeks in SGH with CWD that resulted in an average of >50% weight loss at the time of animal sacrifice when hamsters were still active, but not yet moribund. We have termed this progressive disease in SGH the ‘wasting’ phenotype or WST strain of CWD since overt symptoms of neurological symptoms were not prominent for most of the disease phase. The loss of body mass in WST CWD in SGH was distinct from that reported for other natural prion diseases adapted to SGH including transmissible mink encephalopathy (e.g., HY and DY strains) [27], [28], [31], scrapie (e.g., 263K, 22AH, Me7H strains) [44], [65], CWD [16], [17], bovine spongiform encephalopathy [46], [66], and Creutzfeldt-Jakob disease [48] in which changes in body mass were not evident until the late phases of clinical disease or were not described. Changes in body weight have been reported in sheep scrapie that was adapted to SGH, but in these cases there is a preclinical obesity that is characterized by a >25% increase in body mass for the 22CH and 139H scrapie agent strains [30]–[32], [67]. The cellular basis for either prion-induced cachexia or obesity is not completely understood, but an analysis of energy homeostasis in 139H scrapie in SGH revealed a preclinical hyperphagia, non-fasted hyperinsulinemia with hyperglycemia, and fasted hyperleptinemia that is consistent with an anabolic syndrome that had similarities to type II diabetes mellitus [31], [32]. A similar analysis was performed for HY TME in SGH that exhibit a loss of body mass during the late clinical phase of disease and these animals have a different profile that includes hypersecretion of glucagon, increased fasted ß-ketones, fasted hypoglycemia, and suppressed, non-fasted leptin [31]. It was proposed that in HY TME the SGH had a catabolic syndrome and we would predict that WST CWD in SGH would exhibit a more severe catabolic syndrome than found in HY TME since the weight loss was longer in duration and more pronounced. Additional factors that may have also contributed to the severe loss of body mass in WST CWD in SGH include the increased locomotor activity (e.g., distance traveled, average speed, number of rotations) that was first observed at 43 weeks postinfection and hypophagia that was first recorded at 48 weeks postinfection. These preceded the average time of animal sacrifice at 54 weeks postinfection. This data is suggestive of an increased energy expenditure without an increase in energy intake for several weeks. These prion-induced changes resulted in an imbalance between caloric expenditure and intake and could have exacerbated the loss of body mass.

The observed changes in open field behavior in WST CWD-infected SGH, which proceeded the appearance of clinical signs, are consistent with previous studies that reported increased locomotor activity in rodent prion infections [68]–[70]. The present study confirms and extends these findings by demonstrating preclinical hyperactivity in the CWD SGH model. Increased locomotor activity can represent a hyper-reactivity to novel stimuli or impaired novelty-induced exploration. The detailed analysis of open field behavior in the present study revealed no difference in the thigmotaxis between control and infected SGH subjects, which is indicative of normal emotionality [71]. In contrast, WST CWD in SGH resulted in increased rotational behavior that has not been previously reported. The direction (clockwise vs counterclockwise) of increased rotational behavior observed in experimental hamsters was not consistent and may represent differences in the intra-hemispheric deposition of PrP^Sc^ in brain structures following CWD infection. Although speculative, the wide spread distribution of PrP^Sc^ in structures including the cerebellar white matter, thalamus, hippocampus and substantia nigra can be link to the observed changes in behavior. In addition, abnormal behavior and motor deficits in prion disease models have been associated with changes in the hypothalamo-pituitary-adrenal (HPA) axis [72]. Prion-induced changes in the HPA are also consistent with the wasting syndrome observed in WST CWD in SGH and in cervids with CWD. Irrespective of the origin or mechanism(s), early behavioral markers of prion infection are of epidemiological importance.

Prion-induced endocrinopathies have been proposed to be the primary cause of either obesity or cachexia observed in natural and experimental prion diseases [31], [72]. These can be explained by prion infection of the HPA that results in altered secretion of neuroendocrine hormones or pituitary hormones [31], [72]. These in turn regulate endocrine gland function that can affect body mass and adiposity. Alternatively, prions may directly infect peripheral endocrine glands and alter hormone secretion. Support for these mechanisms can be found in CWD in cervids, sheep scrapie, BSE in cattle, and some human prion diseases in which prion infection has been found in hypothalamic nuclei, the pituitary gland, pancreas, and/or adrenal gland [39], [40], [49], [57], [58], [73]–[76]. In the current study PrP^Sc^ deposition was also found in several hypothalamic nuclei, the pancreas, and adrenal gland in WST CWD in SGH. In both the adrenal gland and pancreas, PrP^Sc^ deposition was associated with markers for neural structures within these tissues suggesting that prion-induced changes in neural control of these endocrine glands could alter hormone secretion. For example, localization of PrP^Sc^ to PGP 9.5-positive structures in the medulla of the adrenal gland could result in a prion effect on sympathetic regulation of adrenaline and noradrenaline, and previous studies have demonstrated increased levels of catecholamines in blood of scrapie infected mice and hamsters [77], [78], and humans with fatal familial insomnia [79]. Similarly, in the pancreas of WST CWD in SGH, PrP^Sc^ deposition was associated with synaptophysin-positive structures in the Islet of Langerhans, which is the region containing the hormone producing cells. In several prion diseases infection of the Islet of Langerhans is reported as well as altered levels of pancreatic hormones in serum including insulin and glucagons suggesting that these endocrinopathies are associated with prion infection of the endocrine system [30]–[32]. In other studies, elevated serum levels of leptin, which acts on the hypothalamus to suppress appetite, was described in 139H scrapie in SGH despite evidence of hyperphagia and obesity [31]. These studies suggest that the dysregulation of the endocrine system in prion diseases is complex and could be due to the targeting of prion infection to multiple sites in both the central and peripheral endocrine system. To understand the cellular basis of wasting disease in CWD additional studies are needed to investigate metabolite and hormone serum levels involved in endocrine function and we propose that the WST CWD in SGH can provide a model to investigate the basis of prion-induced cachexia.

Transmission of CWD from white-tailed deer, mule deer, and elk to TgMo-sghPrP and SGH was undertaken in a previous study [16], and our transmission data of CWD from white-tailed deer into the same founder line of TgMo-sghPrP and outbred SGH produced some different findings. Raymond et al [16] demonstrate a very long incubation period on interspecies transmission of white-tailed deer CWD into TgMo-sghPrP (632±73 days with 4 of 9 mice affected, but 8 of 9 mice were PrP^Sc^-positive in brain) that shortened to 272±62 days on second serial passage, and 212±32 and 237±13 days on third serial passage. Our data resulted in a 100% attack rate and a shorter incubation period on interspecies passage (417±23 days) that stabilized to 198±3 days on second serial passage, and did not significantly shorten on third serial passage (189±5 and 198±3 days). Our transmission data on CWD from TgMo-sghPrP into SGH was also distinct from the previous study [16] in which the mean incubation periods ranged from 408 to 462 days, while in the current study they consistently resulted in mean incubation periods ranging from 322 to 379 days on first and second serial passage into SGH. In passage line B we did observe a longer incubation period (477±15 days) that was consistent with the studies by Raymond et al [16], but this occurred after inoculation of brain material from a CWD infected TgMo-sghPrP that had an incubation period that was 157 days while in the earlier study the inocula was from CWD infected TgMo-sghPrP that had incubation periods significantly longer (>270 days). Therefore, the serial passage history of CWD in TgMo-sghPrP was different between these two studies despite a similar incubation period in a subset of SGH. A comparison of the findings from these two studies may suggest that distinct strains of CWD were isolated upon interspecies passage into TgMo-sghPrP and serial passage into SGH, but these conclusions cannot be reached based solely on incubation periods. More extensive analysis of the biological and biochemical phenotypes of hamster adapted CWD isolates would be necessary in order to identify distinct strains of CWD in SGH. Despite intensive efforts to identify CWD strains using TgMo-cervidPrP, which is a more appropriate model for CWD strain identification, only two distinct strains of CWD have been identified and these are strongly influenced by a normal polymorphism in the cervid *Prnp* [80].

The WST CWD strain in SGH has several similarities to CWD in cervids including a progressive wasting disease, a similar brain distribution of PrP^Sc^, and targeting of prion infection to the neuroendocrine portion of the adrenal gland and pancreas. Additionally, in natural prion diseases of humans and animals, infection of cardiac muscle has only been described in elk and white-tailed deer with CWD [41]. In WST CWD of SGH, PrP^Sc^ was also prominent in heart by western blot and cardiac muscle by immunofluoresence. These findings provide another parallel between CWD infection of cervids and WST SGH. Prion infectivity has been described in muscle from CWD deer [15] and PrP^Sc^ can also be enriched from the tongue of deer and elk infected with CWD [56]. Although low levels of PrP^Sc^ were also described in heart of SGH infected with 263K scrapie and BSE adapted to SGH [66], [81], the levels described in WST CWD appear to be moderately high since more PrP^Sc^ was present in heart than in tongue. Previous studies demonstrate that PrP^Sc^ is not detected in heart while the tongue has higher levels of prion infectivity than other muscle types in 263K scrapie infected SGH [82]. Prion protein deposition in cardiac tissue has also been described in red deer that were experimentally infected with CWD [83]. Other observations such as altered locomotor activity and the distinct distribution of PrP^Sc^ in the olfactory sensory epithelium in WST CWD of SGH have not been directly compared to cervids with CWD in order to determine whether parallel findings are maintained. An advantage of the WST CWD strain in SGH over other rodent models of CWD could be its usefulness in determining the mechanism of prion-induced wasting disease since this key phenotype is maintained for CWD between the deer host and the WST CWD hamster model.

## Materials and Methods

### Animal inoculations, preclinical measurements, and tissue collection

Transgenic mice made from murine PrP null mice were engineered to express the SGH prion protein controlled by the rat neuron-enolase specific promoter as previously described (kind gift of B. Chesebro and R. Race, NIH Rocky Mountain Laboratories, Hamilton, MT, USA) [37], [38]. These mice will be abbreviated throughout this study as TgMo-sghPrP or referred to as transgenic mice since these were the only mice used in the current study. These transgenic mice were estimated to have approximately a four-fold higher level of PrP^C^ in the brain than found in SGH [42]. TgMo-sghPrP were intracerebrally (i.c.) inoculated with 30 µl of a 10% weight per volume (w/v) brain (obex) homogenate from two free range CWD-infected white-tailed deer from Colorado. Brains from these CWD-infected transgenic mice were serially passaged three additional times into additional TgMo-sghPrP over a range of 10^−2^ to 10^−4^ brain dilutions. In addition, brain homogenates from transgenic mice were serially passaged into weanling, Syrian golden hamsters (Simonsen Laboratories, Gilroy, CA) by i.c. inoculation with 50 µl of a 1% w/v brain homogenate. Age-matched mock SGH (i.e., control group) were also inoculated with brain homogenates from normal TgMo-sghPrP or SGH. Following inoculation, individual hamster body weight and cage food consumption were measured on a weekly basis. TgMo-sghPrP and hamsters were observed at least three times per week for the onset of clinical symptoms. Animals were euthanized during the early stages of neurological disease. For collection of tissues for immunohistochemical and immunofluorescence analyses, hamsters were intracardially perfused with periodate-lysine-paraformaldehyde (PLP) fixative, tissues dissected, and processed for embedding in paraffin wax as previously described [33], [34]. For biochemical analysis unfixed tissues were collected, immediately frozen on dry ice, and stored at −80°C until use.

### Locomotor and thigmotaxis activity

Each hamster was individually tested in a standard open field apparatus initially every 14 days (three times) followed by every three weeks for five additional times. The apparatus was a 77 cm square with walls that were 65 cm in height. The floor was covered with paper and then disposed of after each trial. Animals were placed in the center of the apparatus and permitted to explore the environment for three minutes during which activity was recorded using an automated tracking system (ANY-maze, Stoelting, IL, USA). A center, outer, and corner zones were defined and the amount of time and entries into each zone were determined as were the total distance traveled and the average speed.

### Tissue enrichment for PrP^Sc^

Tissues were homogenized in lysis buffer (i.e., 10 mM Tris-HCl, pH 7.4, 150 mM NaCl, 1 mM EDTA, 0.5% deoxycholate, and 0.5% ipegal detergent) to 10% (wt. vol.) using either 0.5 mm glass beads (brain, olfactory bulb, adrenal gland), steel beads (spleen, submandibular lymph node, heart, tongue), or zirconium oxide beads (nasal turbinate) and a Bullet Blender (Next Advance, Averill Park, NY)) at a setting of 6 for 3 minutes. Some tissues were first dissociated using Liberase Blendzyme 2 (Roche Diagnostics, Indianapolis, IN.) in digestion buffer (i.e., Hepes, pH 7.5, 10 mM EDTA) at 55 µg/ml (for the submandibular lymph node and tongue) and 200 µg/ml (for the pancreas) prior to mechanical homogenization with the Bullet Blender. Enrichment for PrP^Sc^ from these tissue lysates was performed following addition of N-lauroylsarcosine, differential centrifugation in a Beckmann Optima™ MAX ultracentrifuge (Beckman Coulter Instruments, Fullerton, CA.), and limited proteinase K digestion as previously described [35]. Proteinase K digestion was performed by resuspending detergent insoluble pellets in TBS (one µl per mg of original tissue weight) and incubating with proteinase K at 10 µg per ml for 30 minutes at 37°C as previously described [35].

### PrP^Sc^ western blot and immunohistochemistry

Tissues enriched for PrP^Sc^ (1 to 50 mg of tissue equivalents per lane) were analyzed on a 12% MOPS NuPAGE gel (Invitrogen, Carlsbad, CA) and proteins were transferred to PVDF membrane. Western blot was performed as previously described using monoclonal anti-PrP 3F4 antibody (gift of V. Lawson, National Institute of Allergy and Infectious Diseases, Rocky Mountain Laboratories, Hamilton, MT), anti-mouse IgG alkaline phosphatase conjugate (Promega Corporation, Madison, WI), and western blots were developed using CDP-Star substrate (Applied Biosystems, Foster City, CA) and imaged with a Kodak Image Station 2000 MM (Eastman Kodak Company, Rochester, NY) [35]. The molecular weight of immunoreactive polypeptides was estimated using the 1D Kodak image software and the Magic Mark protein ladder (Invitrogen Corporation, Carlsbad, CA) were used as standards.

PrP^Sc^ immunohistochemistry (IHC) was performed as previously described [34], [35]. Briefly, animals were intracardially perfused with periodate-lysine-paraformaldehyde (PLP) fixative followed by immersion fixation in PLP (five to seven hours for soft tissues and 24–36 hours for skulls containing the nasal cavity and olfactory bulb). Following immersion fixation, decalcification of skulls was performed by immersion in 10% formic acid as previously described [34], [35]. Once bone was decalcified the nasal cavity, and other tissues, were processed and embedded in paraffin wax. Tissues from a minimum of three animals per group were analyzed. All tissue sections were subjected to antigen retrieval by treatment with formic acid (99% wt. vol.) for 10 minutes. Tissue sections were successively incubated with anti-PrP monoclonal 3F4 antibody overnight at 4°C, then incubated with horse anti-mouse biotinylated secondary antibody (1∶400; Vector Laboratories, Burlingame, CA) at room temperature for 30 minutes, followed by streptavidin-horseradish peroxidase (HRP) at room temperature for 20 minutes. PrP^Sc^ was visualized by localization of HRP activity with DAB+ (DAKO Cytomation, Carpinteria, CA). Tissue sections were counterstained with hematoxylin and cover slip mounted with Mounting Medium (Richard-Allen Scientific, Kalamazoo, MI) for viewing with a Nikon Eclipse E600 microscope. Controls for PrP^Sc^ IHC included the use of mock-infected tissues and substituting a similar concentration of murine IgG isotype control for the anti-PrP 3F4 monoclonal antibody. A minimum of 10 to 30 sections throughout the thickness of the tissue was examined for each animal.

### PrP^Sc^ dual immunofluorescence and laser scanning confocal microscopy (LSCM)

For localization of prion infection to specific cell types or structures, dual immunofluorescence was performed for PrP^Sc^ staining and 1) olfactory marker protein (OMP; goat polyclonal antibody at 1∶750)(Wako Chemicals, Richmond, VA) or adenylyl cyclase III (ACIII; rabbit polyclonal antibody at 1∶1,600)(Santa Cruz Biotechnology, Santa Cruz, CA.) in nasal mucosa, 2) desmin (rabbit polyclonal at 1∶50)(DAKO Cytomation, Carpinteria, CA) in heart, 3) synaptophysin (rabbit monoclonal antibody at 1∶1,600)(Epitomics, Burlingame, CA) in pancreas, and 4) PGP 9.5 (rabbit polyclonal antibody at 1∶2,000)(Biogenesis, Kingston, NH) in adrenal gland. Secondary detection of anti-PrP 3F4 antibody was performed by incubation with horse anti-mouse IgG conjugated to biotin followed by a Alexa Fluor 488 streptavidin conjugate (Invitrogen Corporation, Carlsbad, CA) at a 1∶400 dilution for detecton of PrP^Sc^, and either donkey anti-goat antibody conjugated to Alexa Fluor 594 antibody (1∶800; Invitrogen Corporation, Carlsbad, CA) or goat anti-rabbit antibody conjugated to Alexa Fluor 568 antibody (1∶800; Invitrogen Corporation, Carlsbad, CA) for detection of antibodies to host structures as previously published [36], [62]. The nuclear counterstain ToPro®-3 (Invitrogen Corporation, Carlsbad, CA) was applied to some tissue sections at a 1∶2,000 dilution for 10 min. Tissues from a minimum of three CWD-infected hamsters and three mock-infected animals were examined for each staining procedure.

LSCM images were visualized using a Zeiss LSM 510 Meta confocal system equipped with a Zeiss Plan-Apochromat ×63/NA 1.40 and ×100/NA 1.2 oil objectives. Double immunofluorescence was imaged after excitation of Alexa Fluor 488 with an Argon laser at a wavelength of 488 nm, and excitation of Alexa Fluor 594 with a Helium/Neon laser at a wavelength of 543 nm as previously described [36], [62]. Images were scanned sequentially to minimize crosstalk between channel and the pinhole aperture was adjusted to 1.0 airy units or less for both channels while controlling for identical pinhole diameters and subsequent optical slice thickness. Individual images for stacks were at 0.1 micron per optical slice. Three-dimensional movies were reconstructed from image stacks using the Projection feature of the Zeiss AIM software (Carl Zeiss Microimaging, LLC, North America).

### Statistical Analysis

Behavioral data were evaluated separately using a mixed model ANOVA. In the case of statistically significant interactions, multiple post hoc comparisons were performed using corrected t-tests (Bonferroni's method).

### Ethics Statement

All procedures involving animals and this study were approved by the Montana State University Institutional Animal Care & Usage Committee (Protocol No. 1215) and were in compliance with the *Guide for the Care and Use of Laboratory Animals*; these guidelines were established by the Institute of Laboratory Animal Resources and approved by the Governing Board of the U.S. National Research Council.

## Supporting Information

Video S1 Chronic Wasting Disease in Syrian Golden Hamsters.
(MOV)

Video S2 3D reconstruction of olfactory sensory epithelium in hamster CWD stained for olfactory marker protein and PrP^Sc^.
(MOV)

Video S3 3D reconstruction of olfactory sensory epithelium in hamster CWD stained for adenylyl cyclase III and PrP^Sc^.
(MOV)

Video S4 3D reconstruction of olfactory sensory epithelium from mock hamster stained for adenylyl cyclase III and PrP^Sc^.
(MOV)
